# Angiotensin-Converting Enzyme Inhibitor-Induced Psoriasis: A Case Report on Plaque Exacerbation

**DOI:** 10.7759/cureus.68556

**Published:** 2024-09-03

**Authors:** Abdullah A Almasood

**Affiliations:** 1 General Practice, ABC Medical Center, Riyadh, SAU

**Keywords:** drug-induced psoriasis, plaque psoriasis, psoriasis, angiotensin-converting enzyme inhibitors, hypertension

## Abstract

Psoriasis is a chronic inflammatory skin condition characterized by well-demarcated, erythematous plaques. Certain medications, including Angiotensin-Converting Enzyme (ACE) inhibitors, have been implicated as potential triggers for psoriasis flare-ups. We report the case of a 48-year-old Indian male with a history of well-controlled plaque psoriasis who experienced severe flare-ups after initiating ACE inhibitor therapy for hypertension. Within two weeks, the patient developed widespread psoriatic plaques accompanied by intense pruritus and discomfort, strongly suggesting a drug-induced reaction. Discontinuation of the ACE inhibitor led to a gradual improvement in symptoms, managed with topical corticosteroids and emollients, and switching to an alternative antihypertensive medication resulted in no further exacerbation of psoriasis. This case underscores the potential for ACE inhibitors to trigger psoriasis flare-ups in susceptible individuals, highlighting the need for clinicians to be vigilant when prescribing these medications to patients with a history of psoriasis. Further studies are needed to elucidate the underlying mechanisms and identify patients at risk.

## Introduction

Psoriasis is a chronic inflammatory skin condition characterized by erythematous, scaly plaques that significantly impact patients' quality of life. Affecting approximately 2-3% of the global population, psoriasis is a multifactorial disease with a complex interplay of genetic predisposition, immune system dysregulation, and environmental triggers. Despite advancements in understanding the disease, the exact pathophysiology remains elusive, making it a persistent challenge in dermatology [1].

While the pathophysiology of psoriasis remains complex and multifactorial, one significant factor that can exacerbate or trigger psoriasis is medication use. The connection between psoriasis and drug-induced exacerbations is an area of growing interest and clinical importance. Medications such as beta-blockers, lithium, antimalarials, and nonsteroidal anti-inflammatory drugs (NSAIDs) have long been recognized as potential triggers for psoriasis flare-ups. ACE inhibitors, commonly used to treat hypertension and heart failure, have also been implicated, although their role in psoriasis is less well documented. The immunomodulatory effects of ACE inhibitors may interact with the underlying inflammatory pathways involved in psoriasis, potentially leading to an exacerbation of symptoms. Understanding this interaction is vital for clinicians as it may influence therapeutic decisions, particularly in patients with a known history of psoriasis [2]. However, the role of ACE inhibitors in precipitating or worsening psoriasis is less well documented, making it a critical area for further investigation. Epidemiological data, though limited, suggest that ACE inhibitors may also contribute to psoriasis exacerbations, albeit less frequently than other medications. A recent study utilizing drug-targeted Mendelian randomization and real-world pharmacovigilance analyses has provided additional insight into this potential association [3].

Given the widespread use of ACE inhibitors for managing cardiovascular conditions, the potential for these drugs to trigger psoriasis flare-ups poses significant implications for patient care. This case study not only contributes to the limited literature on ACE inhibitor-induced psoriasis but also highlights the importance of personalized medicine. Recognizing individual patient susceptibilities and monitoring for dermatological side effects can help clinicians better manage the risks associated with ACE inhibitor therapy [4-5].

In this case study, we report the case of a 48-year-old Indian male who experienced severe plaque psoriasis flare-ups after initiating ACE inhibitor therapy for hypertension. The temporal association between the medication and the flare-up, along with the subsequent improvement after discontinuation of the drug, strongly suggests a drug-induced reaction. By examining this case, we aim to expand the understanding of drug-induced psoriasis, reinforcing the necessity for comprehensive drug histories in managing patients with psoriasis and potentially guiding future therapeutic decisions.

## Case presentation

The patient, a 48-year-old Indian male with a longstanding history of well-controlled plaque psoriasis, presented with a significant flare-up of his skin condition shortly after the initiation of lisinopril, an angiotensin-converting enzyme (ACE) inhibitor, for hypertension management. Prior to this, the patient’s psoriasis had been stable for several years, managed with topical treatments. The flare-up began approximately two weeks after starting lisinopril, characterized by the sudden appearance of erythematous, scaly plaques across his trunk and extremities, far exceeding the severity of previous episodes.

**Figure 1:**
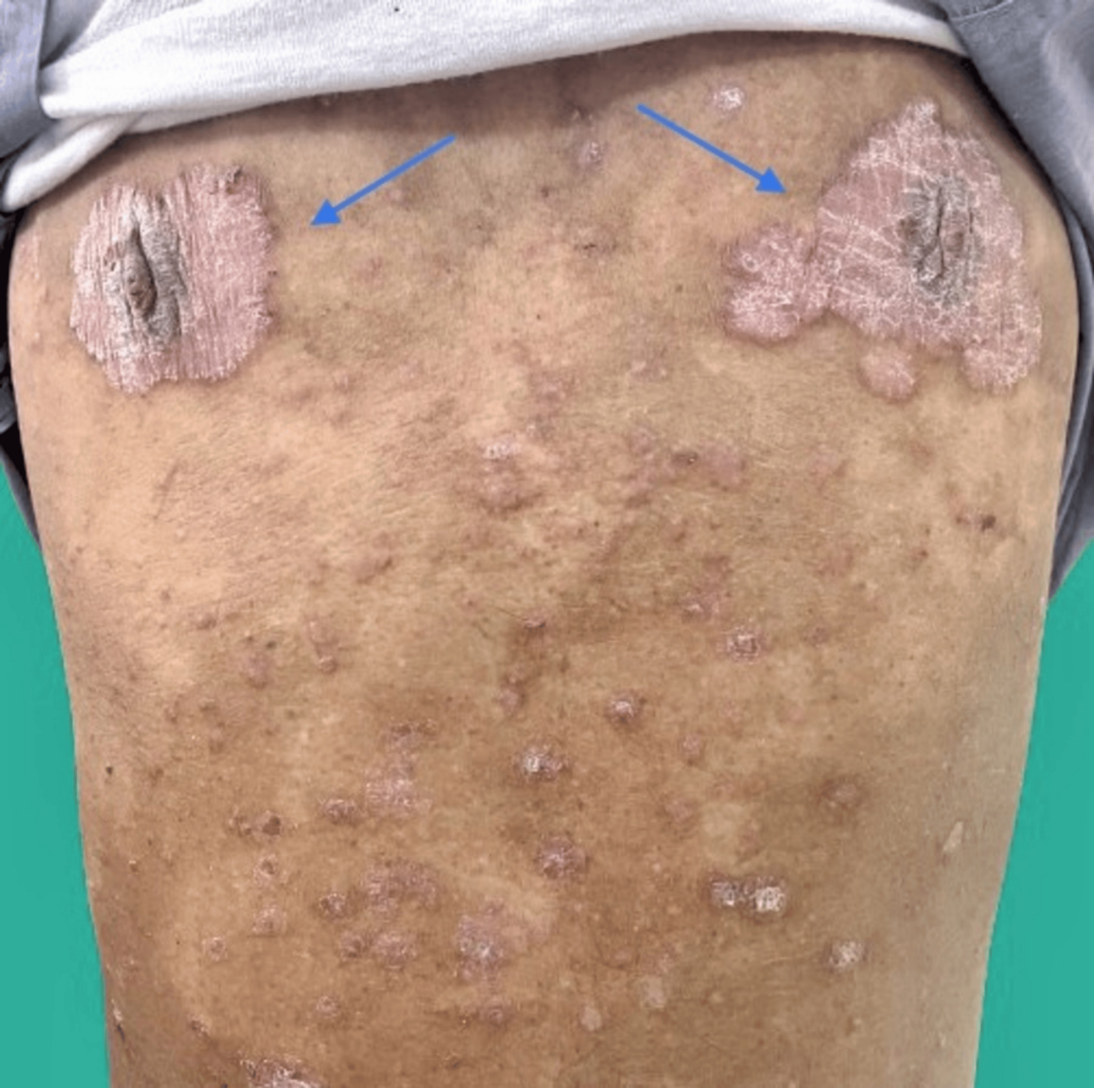
The image shows symmetrical, well-demarcated, erythematous plaques with overlying silver-white scaling, located bilaterally around the nipples. The plaques demonstrate significant thickening, indicating the severity of the flare-up.

Physical Examination revealed symmetrical, well-demarcated erythematous plaques predominantly around the nipples, as well as extensive involvement of the trunk and extremities, with significant scaling and pruritus (Figure 1).

Laboratory tests, including complete blood count (CBC) and C-reactive protein (CRP), were performed to rule out systemic illnesses and infections, such as streptococcal pharyngitis, which is known to precipitate psoriasis flares, and hepatitis (Table 1). These infections were ruled out through a combination of clinical examination and laboratory tests, which showed no signs of active infection. Given the temporal association with the introduction of lisinopril, the ACE inhibitor was suspected to be the primary trigger.

**Table 1:** Laboratory findings of the patient at presentation

Test	Result	Reference Range	Units
White Blood Cell Count (WBC)	8.2	4.0 - 11.0	×10^9/L
Hemoglobin (Hb)	14.8	13.5 - 17.5	g/dL
Platelets	270	150 - 450	×10^9/L
C-Reactive Protein (CRP)	8	<10	mg/L
Erythrocyte Sedimentation Rate (ESR)	10	0 - 20	mm/hr
Blood Glucose	95	70 - 100	mg/dL
Liver Function Tests (LFTs)	Within normal limits	N/A	N/A
Kidney Function Tests	Within normal limits	N/A	N/A

The lisinopril was promptly discontinued, and the patient was treated with a combination of topical corticosteroids and moisturizers, leading to a gradual improvement in his condition over the following weeks. The patient was scheduled for follow-up visits to monitor the progress of his condition and to assess the need for further intervention. At his last follow-up, the patient showed significant improvement with continued topical treatment.

This case underscores the potential for ACE inhibitors to exacerbate psoriasis, particularly in patients with a history of the condition, highlighting the need for careful consideration of medication choices in this patient population.

## Discussion

Psoriasis is a chronic, immune-mediated inflammatory skin condition, typically triggered by a complex interplay of genetic predisposition, environmental factors, and immunological responses. The relationship between medications and the exacerbation of psoriasis has been well-documented, particularly with drugs such as beta-blockers, lithium, antimalarials, and nonsteroidal anti-inflammatory drugs (NSAIDs). However, the role of angiotensin-converting enzyme (ACE) inhibitors, such as lisinopril, in precipitating psoriasis flare-ups remains less clear, possibly due to fewer studies and clinical reports [6]. Interestingly, other ACE inhibitors, including ramipril and perindopril, have also been implicated in psoriasis exacerbations. A case study reported ramipril-induced generalized pustular psoriasis, highlighting the potential for this drug to trigger severe psoriasis flare-ups [7]. Additionally, perindopril has been listed among the drugs responsible for the eruption of psoriasis [8]. These findings suggest that the association between ACE inhibitors and psoriasis may extend beyond lisinopril, making it a topic of emerging clinical interest.

In this case, the sudden and severe exacerbation of plaque psoriasis following the initiation of lisinopril therapy strongly suggests a potential link between the medication and the flare-up. The patient had been stable for several years with no significant psoriasis symptoms until the ACE inhibitor was introduced, indicating a probable drug-induced exacerbation, supported by the temporal association between the drug initiation and the flare-up. While the exact mechanism by which ACE inhibitors might trigger or worsen psoriasis is not fully understood, it is hypothesized that the immunomodulatory effects of these medications may interfere with the inflammatory pathways involved in psoriasis [9].

ACE inhibitors are known to affect the renin-angiotensin system, which plays a role in blood pressure regulation and cardiovascular homeostasis. However, this system also interacts with immune responses, particularly by influencing the activity of T-cells and cytokines such as IL-17, IL-23, and tumor necrosis factor (TNF)-alpha, which are critical components in the pathogenesis of psoriasis. It is plausible that the introduction of lisinopril altered the balance of pro-inflammatory and anti-inflammatory cytokines, exacerbating the patient’s psoriatic condition [10].

Given the widespread use of ACE inhibitors for managing cardiovascular conditions, the potential for these drugs to induce or exacerbate psoriasis has significant implications for clinical practice. This case underscores the importance of personalized medicine, where individual susceptibilities and comprehensive patient histories are carefully considered when prescribing medications. For patients with a known history of psoriasis, alternative antihypertensive agents, such as calcium channel blockers or angiotensin II receptor blockers (ARBs), may be more appropriate, as they have not been similarly associated with psoriasis flare-ups [10].

This case also highlights the need for clinicians to remain vigilant for dermatological side effects in patients receiving ACE inhibitors, particularly those with a personal or family history of psoriasis. Early identification and intervention can prevent severe exacerbations, ensuring better patient outcomes. Further research is needed to elucidate the precise mechanisms by which ACE inhibitors may induce psoriasis and to explore whether certain patient populations are more at risk than others. Comparative studies between ACE inhibitors and other classes of antihypertensive drugs could provide valuable insights into safer alternatives for psoriasis patients.

In conclusion, while the connection between ACE inhibitors and psoriasis flare-ups is not yet fully established, this case contributes to the growing body of evidence suggesting a potential link. Clinicians should be aware of this association and consider it when managing hypertensive patients with psoriasis to minimize the risk of drug-induced exacerbations. Continued research and clinical awareness are vital to developing evidence-based guidelines that ensure the safe and effective treatment of patients with both hypertension and psoriasis.

## Conclusions

This case underscores the potential for ACE inhibitors, such as lisinopril, to exacerbate psoriasis in susceptible individuals. The sudden flare-up of plaque psoriasis in a patient with a stable history following the initiation of an ACE inhibitor highlights the importance of considering patient history when prescribing these medications. While ACE inhibitors are effective for managing hypertension and heart failure, their potential to induce or worsen psoriasis should not be overlooked, particularly in patients with a known predisposition to the condition.

The exact mechanism behind ACE inhibitors precipitating psoriasis flare-ups is not fully understood. It is hypothesized that these medications may influence immune system pathways, leading to increased inflammatory responses, possibly through the modulation of angiotensin II and its effects on cytokine production. Clinicians should exercise caution when prescribing ACE inhibitors to patients with a history of psoriasis, consider alternative antihypertensive therapies when appropriate, and closely monitor those who require ACE inhibitors to ensure early detection and management of any flare-ups. This case highlights the need for further research into the relationship between ACE inhibitors and psoriasis, contributing to the ongoing discourse on drug-induced dermatological conditions and emphasizing the value of personalized medicine in optimizing patient care.
